# Notes

**Published:** 1899-09-09

**Authors:** 


### NOTES.

The Japanese warship "Yikishima," lying at the Royal Albert Docks, has been thrown open to visitors at a small charge for the benefit of the Seamen's Hospital. A few years ago the Japanese authorities allowed their ship "The Fuji" also to be visited in the same manner in aid of the hospital.

---

In the new Nottingham workhouse, now in course of erection, the care of the sick has received most careful consideration, and excellent and well-arranged accommodation will be provided. The various cases are to be carefully divided, and isolation wards, a small hospital for epidemics containing male and female wards, and a ward for children are being constructed. The staff also will be excellently housed.

---

In the latest of the fever hospitals opened by the Metropolitan Asylums Board—the Grove Hospital at Tooting—the wards are arranged *en échelon*, a plan which, although it has been adopted in various foreign hospitals, is far from common in England. No doubt the architect's object has been to utilise the ground to the best advantage, while retaining the advantage of getting the sun upon both sides of the wards. In any case the general result is very good. It will be remembered that the wards on the deck of the old "Castalia" small-pox ship were arranged in somewhat the same way.

---

The last quarterly meeting of the Middlesex Hospital was marked by the discussion of matters of more than usual interest. Foremost amongst these was the report of the accusation which had been brought against the authorities by the *Evening News* of concealing a case of plague. The injury was aggravated by the fact that, although the editor of the paper in question had stated that he was prepared to prove the charge, on investigation being requested the whole matter was allowed to drop, although the charge was not withdrawn. At the meeting the secretary superintendent, Mr. Clare Melhado, reported that the cancer wards were progressing rapidly, but it was not intended to open them until special laboratories were ready for the purpose of study and research in this branch of disease.

---

The hospital at Assouan, constructed by Messrs. John Aird for the use of their workpeople employed in constructing the Nile Reservoir, has developed into quite an important institution. It commenced on quite a small scale, and as the demands upon its resources increased so were beds added, until now there is accommodation for nearly 50 patients. The hospital consists of a series of separate buildings, two pavilions being devoted to Europeans, one to natives, besides which there are three more for such infectious cases as may occur. Since October last year 198 cases have been received, 28 of which proved fatal. Of the 33 cases of heat affections (sunstroke, &c.) all but one were among Europeans. Ten of these cases died, but in eight of them death took place before medical aid could be rendered. The hospital is in charge of Dr. Schmitt, who has two assistant medical men, three male nurses, eight orderlies, and a female housekeeper on his staff.

